# New Perspectives on DNA and RNA Triplexes As Effectors of Biological Activity

**DOI:** 10.1371/journal.pgen.1005696

**Published:** 2015-12-23

**Authors:** Albino Bacolla, Guliang Wang, Karen M. Vasquez

**Affiliations:** Division of Pharmacology and Toxicology, College of Pharmacy, The University of Texas at Austin, Dell Pediatric Research Institute, Austin, Texas, United States of America; Stanford University School of Medicine, UNITED STATES

## Abstract

Since the first description of the canonical B-form DNA double helix, it has been suggested that alternative DNA, DNA–RNA, and RNA structures exist and act as functional genomic elements. Indeed, over the past few years it has become clear that, in addition to serving as a repository for genetic information, genomic DNA elicits biological responses by adopting conformations that differ from the canonical right-handed double helix, and by interacting with RNA molecules to form complex secondary structures. This review focuses on recent advances on three-stranded (triplex) nucleic acids, with an emphasis on DNA–RNA and RNA–RNA interactions. Emerging work reveals that triplex interactions between noncoding RNAs and duplex DNA serve as platforms for delivering site-specific epigenetic marks critical for the regulation of gene expression. Additionally, an increasing body of genetic and structural studies demonstrates that triplex RNA–RNA interactions are essential for performing catalytic and regulatory functions in cellular nucleoprotein complexes, including spliceosomes and telomerases, and for enabling protein recoding during programmed ribosomal frameshifting. Thus, evidence is mounting that DNA and RNA triplex interactions are implemented to perform a range of diverse biological activities in the cell, some of which will be discussed in this review.

## Introduction

In the past decade, advances in the field of DNA structure and in the genetic and biological functions of its polymorphic conformations have led to the important realization that DNA is not simply a passive carrier of genetic information. Rather, by adopting conformations that differ from the canonical B-form DNA double helix, the DNA itself plays active roles in cellular processes. Knowledge that DNA bases can engage in hydrogen bonding interactions that differ from the canonical Watson-Crick bonding patterns, and that DNA strands are able to form secondary structures that deviate from the common (B-form) right-handed double helix, dates back to the early 1950s [1,2]. Since that time, more than a dozen such alternative DNA conformations, collectively called non-B DNA, have been characterized. Parallel to this pioneering work, sequencing efforts predating the draft of the human genome sequence clearly revealed that non-B DNA-forming motifs are strongly overrepresented in mammalian and other genomes, and that non-B DNA structures form in vivo, spurring interest in the question of their potential biological function [3,4].

In 1991, the discovery of a new class of hereditary neurological diseases caused by the expansion of unstable microsatellite repeats marked a turning point in the field of non-B DNA, with the research that followed firmly establishing a direct connection between the formation of noncanonical DNA structures in genomes and human disease [5–8], predominantly mediated by DNA repair mechanisms [9–12]. In more recent years, the field has diversified considerably, in part due to the realization that, as a consequence of widespread transcriptional activity genome-wide, the opportunities for RNA and DNA–RNA interactions leading to complex nucleic acid secondary structures is enormous. Herein, we provide an update on DNA–RNA and RNA triplex structures, with an emphasis on their emerging roles as effectors of biological activity, and refer to recent reviews on DNA triplexes [10,13,14].

## Triplex Interactions

**Fig 1:**
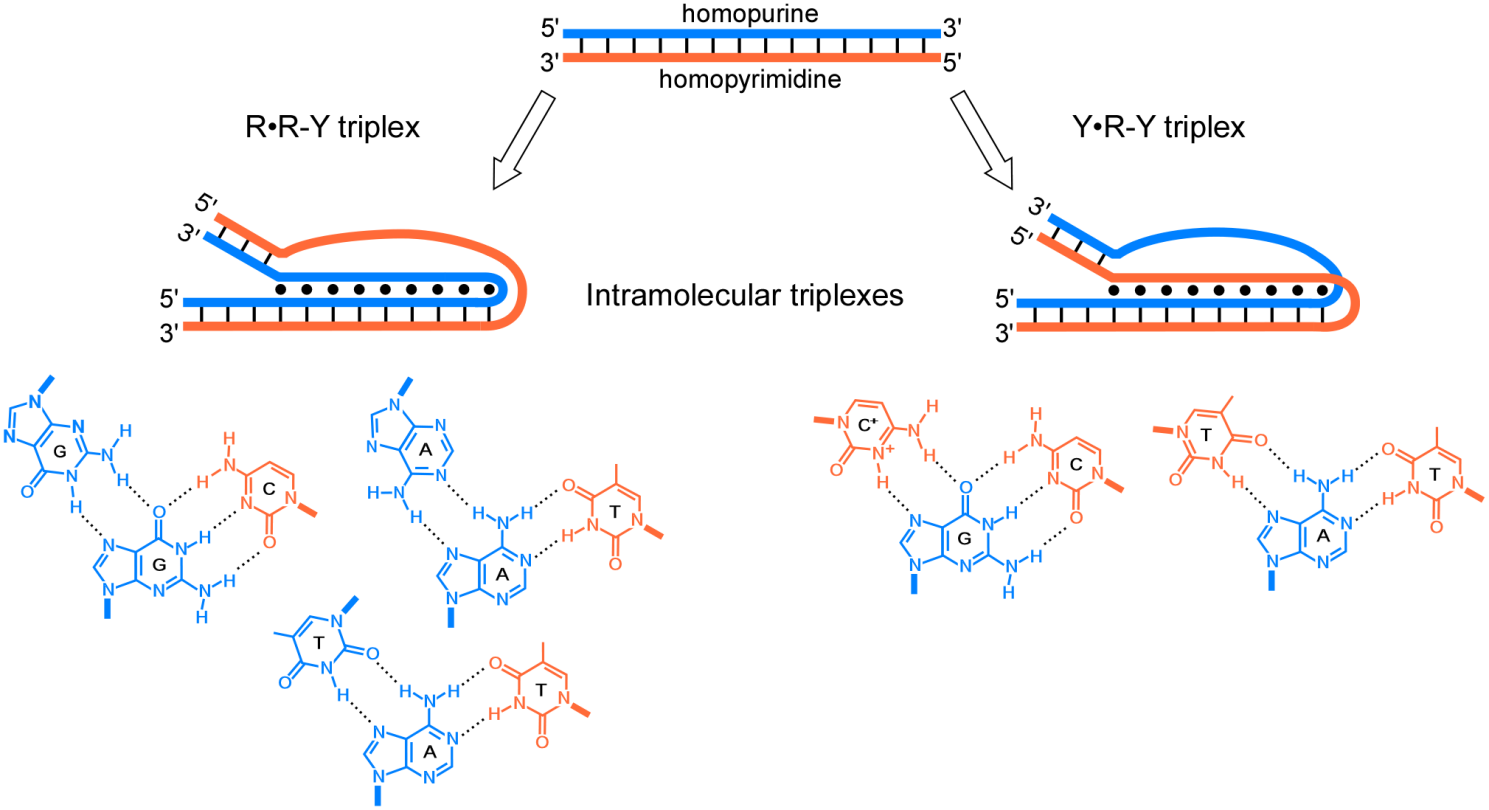
Model of intramolecular DNA triplexes and common triplets. In genomic DNA in vivo or in supercoiled plasmid DNA in vitro, homopurine–homopyrimidine tracts with mirror-repeat symmetry, i.e., AGGAA…AAGGA-TCCTT…TTCCT (top) may form four types of triplex structures: two in which half (either the 5′ or 3′ half; only the 3′ case is shown) of the single-stranded purine-rich strand folds back, engaging in reverse Hoogsteen interactions with the purine-rich strand of the remaining duplex in an antiparallel orientation (R•R–Y type triplex, left); and two in which half (either the 5′ or 3′ half; only the 3′ case is shown) of the single-stranded pyrimidine-rich strand folds back, engaging in Hoogsteen interactions with the purine-rich strand of the remaining duplex in a parallel orientation (or Y•R–Y type triplex, right). The most common triplets, both in intramolecular and intermolecular (triplex-forming oligonucleotide [TFO]-derived) triplexes, include A•A–T, G•G–C, and T•A–T for R•R–Y type triplexes (bottom left), and C^+^•G–C and T•A–T for Y•R–Y type triplexes (bottom right). C^+^ indicates a protonated cytosine.

Triple-helical nucleic acid interactions have been characterized by a variety of techniques on oligonucleotides and plasmid DNA [2,3]. Triplex interactions are mediated at homopurine–homopyrimidine sequences with mirror repeat symmetry by Hoogsteen hydrogen bonding between the purine-rich strand of duplex DNA and either a pyrimidine-rich or a purine-rich third strand (Fig 1). Pyrimidine-rich third strand interactions are stabilized by T•A–T and C^+^•G–C Hoogsteen hydrogen bonds (“•” = Hoogsteen hydrogen bonds; “–” = Watson-Crick hydrogen bonds; R = A or G; Y = C or T) and are particularly favored at low pH, which facilitates the requirement for cytosine protonation at the N3 position (Fig 1, right). By contrast, purine-rich third strand interactions form A•A–T and G•G–C reverse-Hoogsteen hydrogen bonds (Fig 1, left), which do not require acidic pH but are stabilized by bivalent cations such as Mg^2+^.

**Fig 2:**
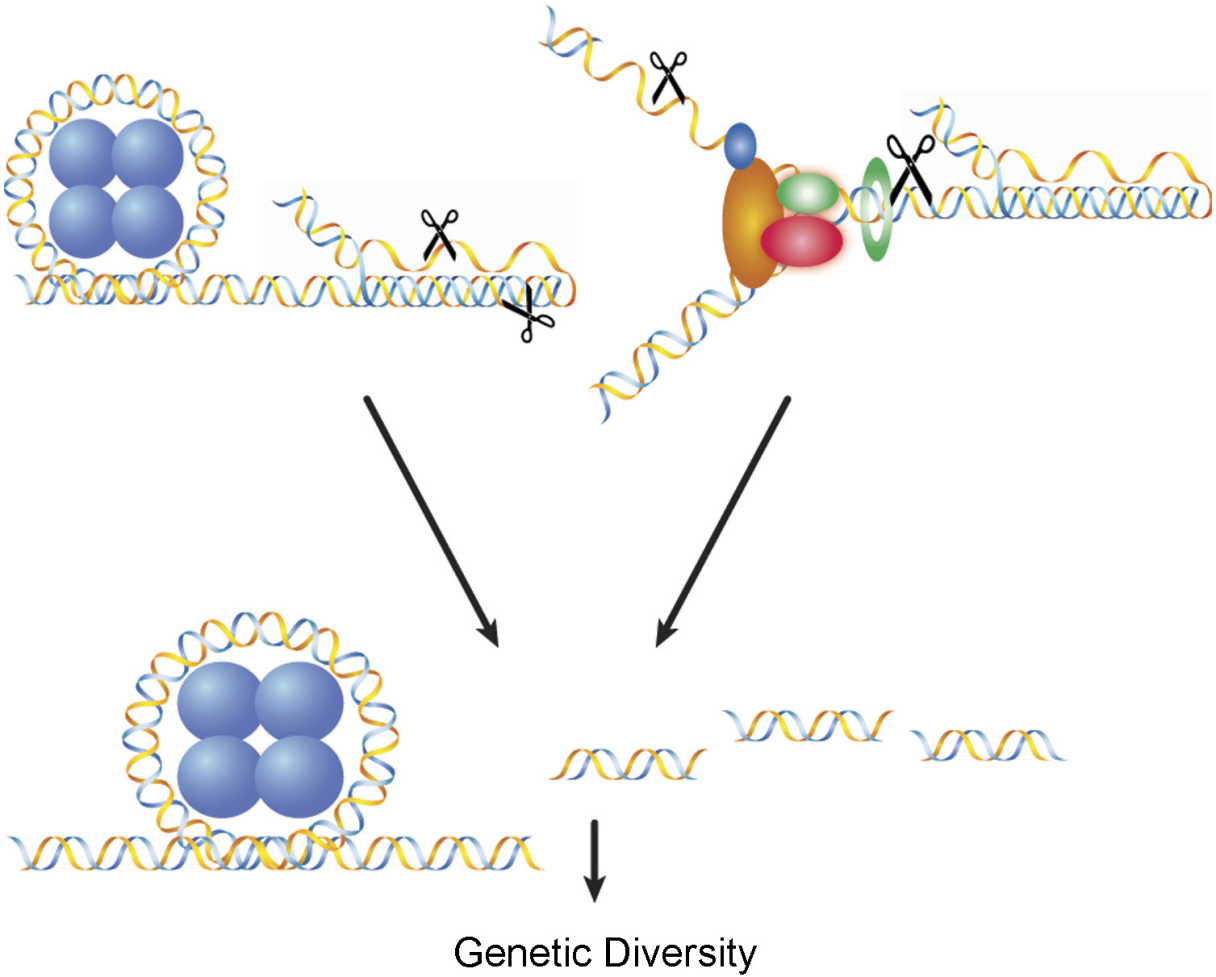
Model of DNA triplex-induced mutagenesis and genomic diversity. DNA repair proteins (shown as scissors) recognize and process DNA triplex structures in replication-independent (left) and replication-related (right) pathways, and may contribute to genomic instability and, perhaps, genomic diversity.

Triplex DNA-forming sequences have been implicated in a wide array of biological activities, including gene expression regulation [15–17], replication pausing [18–20], and genetic instability leading to human disorders, including cancer [6,7,21–26]. In the context of genomic instability, human triplex-forming DNA sequences integrated into the mouse genome underwent higher rates of large deletions, while no instability was detected in control B-DNA-forming sequences [22]. A number of reports suggest that triplex DNA may elicit genetic instability via several mechanisms, such as serving as a roadblock to DNA replication and transcription elongation [25,27–29]. Replication-independent models have also been suggested; for example, the helical distortions and structural alterations induced by triplex DNA may be recognized as “DNA damage” and subsequently processed in an error-generating fashion (Fig 2) [23].

## Roles of Noncoding RNA in Biologically Active DNA–RNA Triplexes

The recent discovery that transcription is not limited to protein-coding genes in the human genome, but is a widespread activity taking place across most (approximately 80%) of chromosomal DNA, yielding families of noncoding RNAs, has raised awareness of pivotal cellular functions played by interactions between noncoding RNAs and proteins, protein-coding RNA transcripts, and genomic DNA [30–32]. The most common types of noncoding RNA include microRNAs, short (22 nt) RNAs that inhibit protein synthesis by binding to specific mRNAs, long (>200 nt) noncoding RNAs (lncRNAs), a number of which have been associated with the regulation of gene transcription, splicing, and translation [33], and small nucleolar RNAs, which participate in chemical modifications of other RNAs, including ribosomal (rDNA) and transfer RNAs (tRNA).

**Fig 3:**
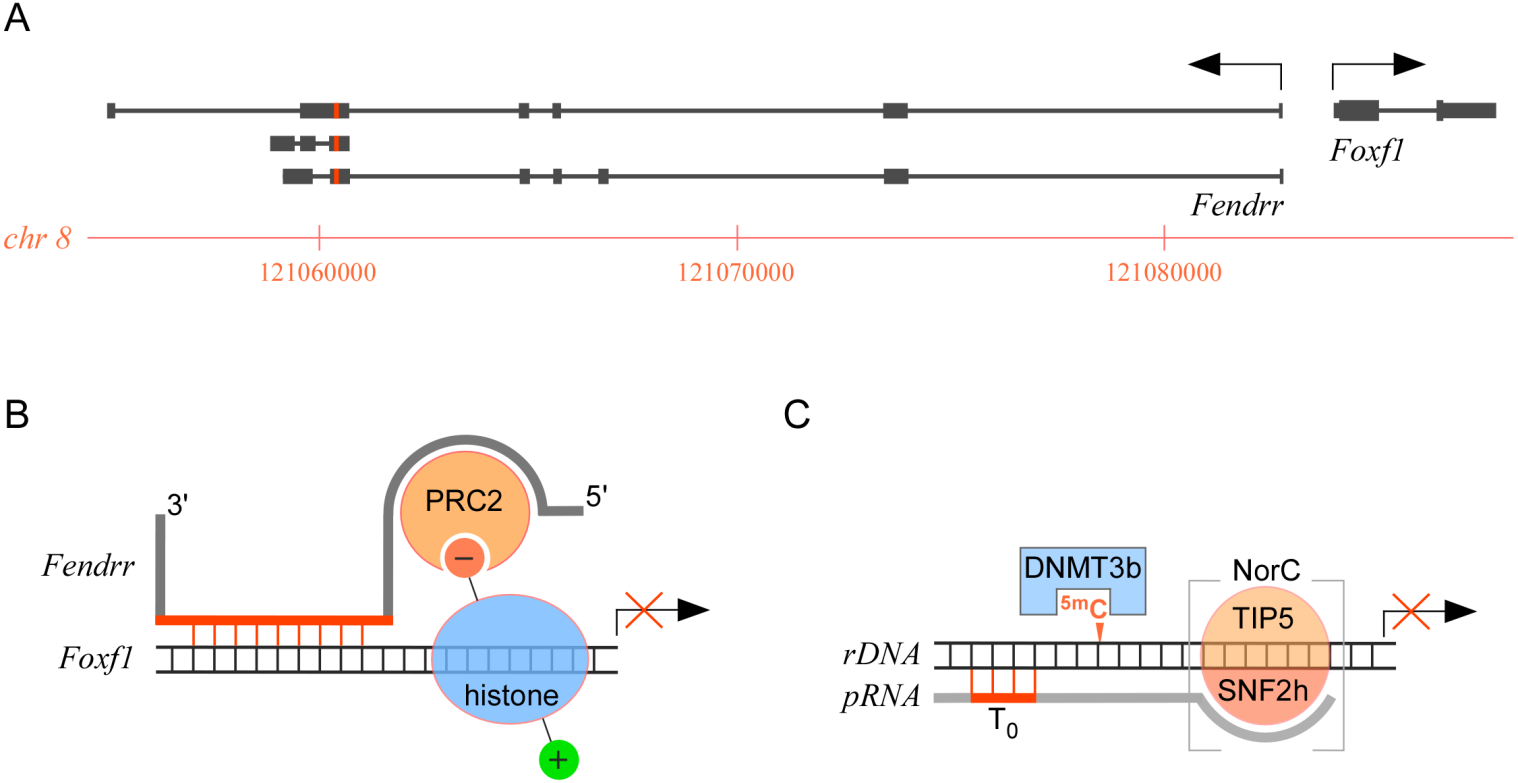
Noncoding RNAs achieve gene regulation through triplex interactions. (A) Illustration of the organization on the mouse genome of the *Fendrr* and *Foxf1* genes. The *Foxf1* gene comprises two coding exons (large rectangles) separated by a short intron (small rectangle), and 5′ and 3′ untranslated exons (medium rectangles). Different splice variants have been identified for the *Fendrr* gene, which include lncRNA transcripts (medium rectangles) that contain a sequence involved in triplex interactions with *Foxf1* and other genes (red). The two genes are transcribed in the opposite orientation (arrows) from a shared promoter. (B) Diagram showing the scaffolding role of *Fendrr* lncRNA, achieving gene regulation by anchoring to target genes (*Foxf1*) through triplex interactions with cognate duplex sequences (red lines), and delivering chromatin modifiers (PRC2) to key histone tail residues (red circle). (C) Illustration of rDNA gene silencing by noncoding RNAs binding to T_0_ through triplex interactions at one end and to the nucleolar remodeling complex (NorC) silencing complex at the opposite end, and by cytosine methylation catalyzed by DNA cytosine-5-methyltransferase 3b (DNMT3b).

The association between noncoding RNA transcripts and genomic DNA is particularly relevant in the context of this review due to the formation of biologically active RNA–dsDNA triplex structures [34]. One such structure, described at the mouse *Foxf1* locus, is thought to serve a scaffolding role for the delivery of site-specific epigenetic modifications leading to gene silencing. Specifically, divergent transcription from the *Foxf1* promoter generates an lncRNA, termed *Fenddr*, whose expression is critical for embryonic development in mice. *Fendrr* has been shown to bind specifically to the epigenetic modifying system polycomb repressive complex 2 (PRC2), a protein complex that carries out trimethylation of histone 3 lysine 27 residues (H3K27me3), which in turn inhibits transcription. Target genes for the inhibitory activity of the *Fendrr*-PRC2 complex include the *Foxf1* gene itself and *Pitx2*, which is critical for the development of internal organs. *Fendrr* RNA is believed to carry out two separate activities: (1) binding to duplex promoter sequences to form an RNA–dsDNA triplex; and (2) anchoring the PCR2 complex (Fig 3A and 3B). *Fendrr* also binds other molecules, such as the trithorax group protein TrxG/Mll, which sets active transcription marks by methylating lysine 4 residues, also on histone H3 (H3K4me3). Hence, triplex formation appears to serve a general scaffolding role, irrespective of the enzymatic function of the cognate complexes. Indeed, scaffolding is emerging as a common role for lncRNAs, such as the HOX transcript antisense RNA (HOTAIR, also a partner of PRC2), which is overexpressed in a number of cancers, including primary and metastatic breast tumors [35,36].

A role for noncoding RNA–dsDNA triplex formation in gene silencing has also been suggested for ribosomal RNA-encoding (rDNA) genes [37]. In mammalian genomes, only a subset of the tandem arrays of rDNA genes is transcribed, with silencing being achieved by a combination of repressive chromatin marks and methylation of a pivotal CpG site at the rDNA promoter [38]. The heterochromatin state is maintained by the nucleolar remodeling complex NorC, which includes the TTF-I-interacting protein 5 (TIP5) and SNF2h, and a noncoding RNA transcribed from the rDNA promoter itself (pRNA). The current model for rDNA gene silencing suggests a critical role for pRNA, which interacts with the TIP5-SNF2h complex and forms an RNA–dsDNA triplex with the rDNA promoter region [37]. The RNA–dsDNA triplex structure is proposed to elicit gene silencing in three ways: first, by occluding T_o_, a critical binding site for the TTF-I transcriptional activator; second, by recruiting the DNA cytosine-5-methyltransferase DNMT3b to methylate the pivotal CpG site; and third, by recruiting histone deacetylases and histone methyltransferases through the TIP5-SNF2h complex to consolidate a heterochromatin structure (Fig 3C) [37].

A thought-provoking hypothesis has recently emerged from work on lentiviral-infected cells, which suggests a critical role for microRNAs in maintaining viral latency through RNA–dsDNA triplex-formation [39,40]. Based on a number of findings, including the key observation that HIV-1 viral loads in peripheral blood mononuclear cells of HIV-1-infected individuals correlate negatively with immunofluorescence staining for triplex nucleic acids, the authors propose that one of the roles of microRNAs is to counteract viral infection. The model envisions that in primates, microRNAs synthesized from transposons, retroviruses, and other retro-elements form RNA–dsDNA triplexes with appropriate homopurine–homopyrimidine regions of the proviral genomes in the cytoplasm, thereby preventing migration, integration, and viral replication in the nucleus [40].

These composite data open new frontiers into the roles of noncoding RNAs as biological effectors of functional, high-order nucleic acid secondary structures in the cell. Nevertheless, further studies are needed to clarify the details of interactions between noncoding RNAs and their duplex DNA target sites.

## RNA Triplexes

Reports that two to six consecutive base triplets may form in RNA, either by the folding of a single molecule or by the interaction between two RNA molecules or RNA ligands, have been present in the literature since the late 1970s [41]. However, the notion that RNA triplexes occupy a significant niche in various biological processes is only recently being realized. Of note, intramolecular triplex formation has been shown to dramatically stabilize some lncRNAs, such as human MALAT1, one of the most abundant and highly conserved lncRNAs, which functions as a cis-factor in gene expression regulation [42,43], and polyadenylated nuclear RNA from Kaposi's sarcoma-associated herpesvirus [44]. Here, we review progress made on RNA triplexes with a focus on work published during the past few years, and refer the reader to recent reviews [45,46] and specific publications on related riboswitches [47] and structural RNA motifs [48].

## An RNA Triplex at the Catalytic Center of Spliceosomes

**Fig 4:**
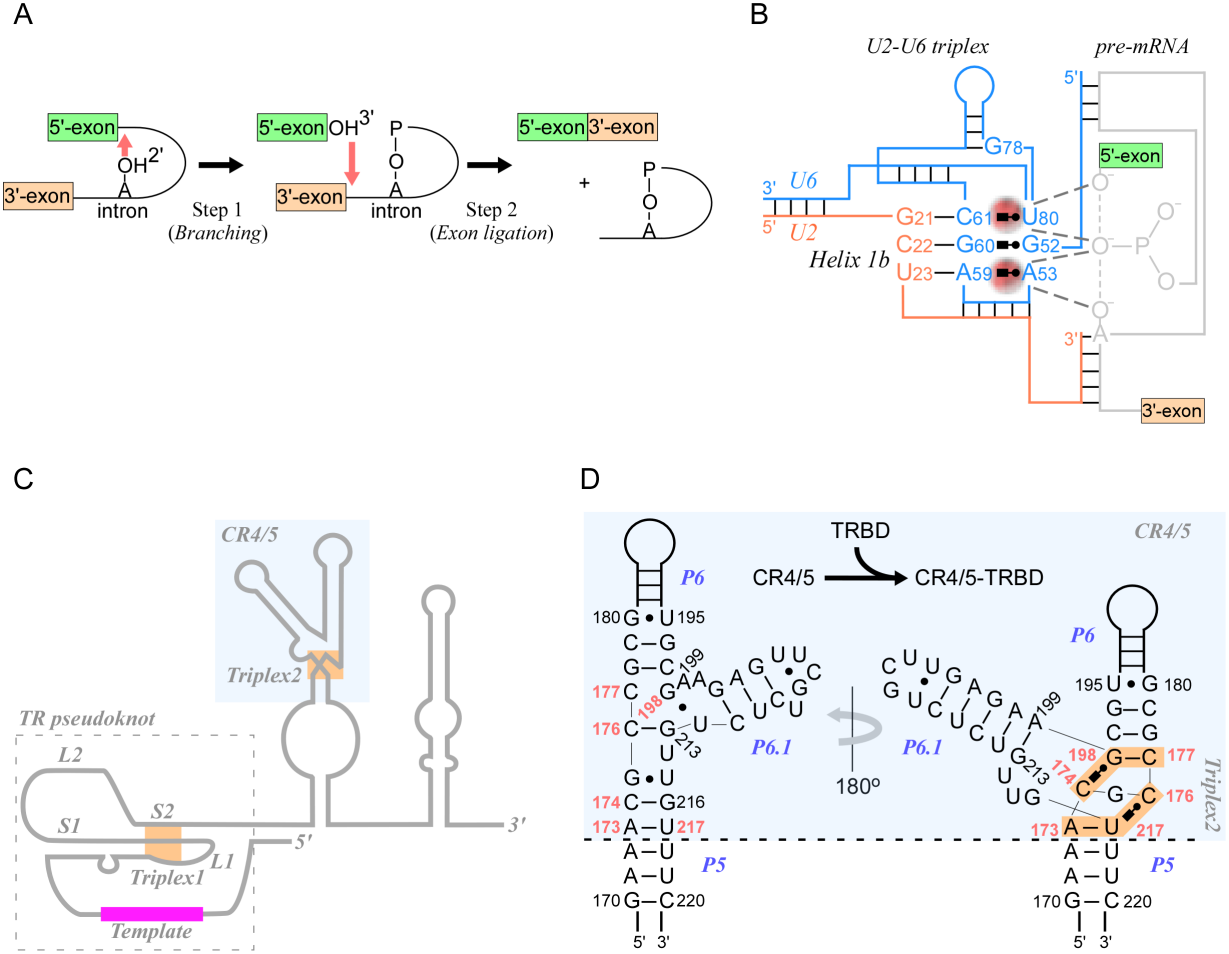
RNA triplexes perform critical functions in biological systems. (A) Schematic of the two catalytic steps of splicing. In the first step (branching), the 2′-hydroxyl of an intronic adenosine (branch point) attacks the phosphodiester bond at the 5′-exon-intron boundary, releasing the 5′-exon with a free 3′-hydroxyl and a lariat structure comprising the 5′-intronic phosphate group linked to the 2′-hydroxyl of the attacking adenosine. In the second step (exon ligation), the 3′-hydroxyl of the free 5′-exon attacks the 3′-intron-exon boundary, thereby releasing the intron lariat and the fused 5′-to-3′-exons. (B) Illustration of the triplex formed by U2-U6 RNAs of yeast spliceosome. Bases from U6 (blue) and U2 (orange) create a triplex structure that coordinates two magnesium ions (red) required for both steps of catalysis on pre-mRNAs (gray). The first catalytic reaction is shown, i.e., attack of the 2′-OH of an intronic adenosine on the 5′-exon-intron junction phosphate group. (C) Outline of the TR component of telomerase (medaka), displaying the core pseudoknot region comprising two loops (L1 and L2) and two stems (S1 and S2), template (red), and two triplexes (orange), one at the pseudoknot and the other at the CR4/5 domain (blue shading). (D) Close-up of the CR4/5 domain showing base-pair interactions in the absence (left) and in the presence (right) of the TR-binding domain (TRBD). TRBD binding reorganizes critical bases (red) at the junction between P5, P6, and P6.1 and form a mini-triplex, causing P6.1 to rotate by approximately 180° (blue shading).

A significant advance related to RNA triplexes is the recent discovery of a catalytic role in RNA splicing, the key cellular mechanism through which introns are removed from pre-mRNA. Recently published genetic and biochemical studies suggested that RNA triplex formation within a spliceosome promoted catalytic metal binding and the consecutive cleavage steps (Fig 4A) [49]. Spliceosomes, large ribonucleoprotein machineries comprising approximately 170 proteins in humans and five small nuclear RNAs (U1, U2, U4, U5, and U6 snRNAs), assemble into dynamic complexes to enable splicing [50]. Strong similarities in sequence conservation and folding intermediates between the U6 snRNA subunit of spliceosomes and domain V of group II introns [51], transposable elements encoding RNAs that self-splice in the absence of proteins, have contributed to longstanding speculation that spliceosome catalysis is RNA-based [52–54]. Leveraging on the crystal structure of *Oceanobacillus ihejensis* group II introns [55], in which an RNA triplex coordinates two magnesium ions essential for catalysis, metal rescue experiments, genetic mutation analyses, and cross-linking assays have shown that both steps of splicing require a triplex structure formed by the RNA components of the spliceosome [56,57]. In yeast, a highly conserved 5′-AGC-3′ triad in helix 1b of U6 base pairs with both 5′-GCU-3′ residues of U2 through canonical Watson-Crick hydrogen bonds, and through tertiary interactions with distant bases in U6 and a conserved bulged U80, to yield three triplets (Fig 4B). Residues U80, A59, and G78 engage in direct contact with two magnesium ions that assist both catalytic steps of pre-mRNA cleavage [56,57].

Although a high-resolution structure of the spliceosome catalytic site is not yet available, the analogy with group II introns supports the view that both machineries function as ribozymes and share a common evolutionary ancestor. In the crystal structures of *O*. *ihejensis* and the brown algae *Pylaiella littoralis* group II introns, the triplex structures serve to create a geometrical and negatively charged cage for the recruitment of two metal ions and their placement at a critical distance of approximately 3.9 Å from one another. This arrangement enables classic two-metal ion catalysis [58], a mechanism commonly found in enzymes such as DNA and RNA polymerases [52]. A number of questions remain, including whether the same triplex structure operates during both steps of catalysis, and whether the protein scaffold induces triplex formation and mediates its interactions with the pre-mRNA [59–61].

## Two RNA Triplexes Are Required for Telomerase Activity

Telomeres, specialized DNA-protein complexes at the end of linear chromosomes, protect chromosomes from end-to-end fusion and erosion. Their length is maintained by telomerase, a ribonucleoprotein complex that adds species-specific DNA repeats by using a reverse transcriptase activity (TERT) on an internal RNA component (TR) [62]. At the core of TRs is a pseudoknot (Fig 4C), a common RNA fold [48] comprising two loops and two stems, which, in addition to duplex interactions, is further stabilized by a triplex structure that has been shown to confer optimal telomerase activity [45]. Base triples in TR pseudoknots have also been predicted in ciliates [63], and a recent investigation on the yeast *Kluyveromyces lactis* has confirmed the structural similarities with the human TR triplex and its requirement for telomerase activity [64]. NMR and mutation analyses support the formation of an extended pyrimidine-rich triplex in *K*. *lactis* telomerase TR. Key issues that remain to be resolved include the role of TR triplexes in telomerase activity, how the triplex structures contribute to catalysis, and whether geometrical arrangements imposed by the triplexes also play a role in catalysis [45]. Nevertheless, the finding that triplexes are in close proximity to the catalytic center and the template has been taken as an indication that triplex structures in telomerases are essential for function in vivo [64].

A second 2-triple minihelix has recently been reported in the CR4/5 subdomain of the vertebrate *Oryzias latipes* (Japanese medaka) TR in complex with the TR-binding domain (TRBD) of TERT (Fig 4C and 4D) [65]. These and other TRBD-induced changes occur at a three-way junction between helices P5, P6, and P6.1, causing P5 and P6 to stack coaxially and P6.1 to rotate by over 180° around the junction region and onto TRBD (Fig 4D). However, the precise role of this two-tier triplex and the associated CR4/5 conformational changes in telomerase function remains unresolved. For example, A199, a highly conserved residue at the P6/P6.1 junction, forms a noncanonical pair with G213 in CR4/5-TRBD, but it is unpaired in free CR4/5 (Fig 4D) [65,66]. Mutation analyses at A199 and at the corresponding residue in *Schizosaccharomyces pombe*, *Neurospora crassa*, and humans have produced conflicting results by either disrupting CR4/5 interactions with TRBD and impairing telomerase function [65] or by causing minor defects [66].

## RNA Triplexes Serve As Roadblocks That Promote Recoding

Programmed ribosomal frameshifting (PRF) refers to the property observed in several viral mRNAs of generating alternative reading frame proteins through recoding, whereby ribosomes are forced to shift by +/- 1 or +/- 2 bases to continue translation [67]. The mRNA signals that induce -1 PRF include a “slippery” sequence, such as UUUAAAC, UUUUUUA, etc., followed by a stimulatory structure (a physical barrier often represented by a pseudoknot). The “slippery” sequence and physical barrier are believed to act in concert to pause the ribosome over the repetitive sequence, stimulating its repositioning on alternative reading frames before resuming translation. In some viruses, such as the Beet western yellows virus, Pea enanion virus type-1, Sugarcane yellow leaf virus, and Simian retrovirus type-1, the pseudoknots are stabilized by triplex interactions [68], similar to the TR telomerase described above. In the absence of such ternary RNA interactions, PRF is inefficient, and mutational analyses support the view that triplex structures serve to increase pseudoknot stem stability and torsional resistance, both of which strengthen the mechanical obstacle to mRNA translocation on ribosomes [69,70].

Mounting evidence supports the view that -1 PRF is not limited to viral mRNAs. Aside from the finding that approximately 10% of genes in eukaryotic genomes are predicted to contain -1 PRF signals and that -1 PRF controls telomerase maintenance in yeast [71], a novel mechanism for -1 PRF based on the interaction between microRNAs and pseudoknots has recently been reported in human cytokine receptor mRNAs [72]. In the specific case of CCR5, miR-1224 is thought to form a triplex with the CCR5 mRNA pseudoknot and to promote -1 PRF. Since this PRF is followed by mRNA degradation through the nonsense-mediated mRNA decay pathway, it is believed to represent a mechanism for regulating the cellular response to cytokines [72]. Direct evidence for miR/pseudoknot triplex structures requires further study. Nevertheless, the finding that PRF signals are common in eukaryotic genomes and that noncoding RNAs, an abundant pool of genomic transcripts, may participate in triplex interactions either with RNA or DNA [37,38,73,74] suggests that RNA triplex interactions in genomes may occur more frequently than currently appreciated.

## Conclusions

It has become increasingly recognized that triplex DNA, as well as other non-B DNA structures not discussed herein, such as quadruplex DNA, cruciforms, slipped structures, and left-handed Z-DNA, are an intrinsic source of genetic instability within the cell ([10,75] and references therein). This is an emerging novel concept, since the conventional view has been that genetic instability results from insults to the DNA from extrinsic factors (e.g., oxidants, ultraviolet light, carcinogens) and faulty DNA repair. A main area of investigation for the future will be to identify the cellular pathways that recognize non-B DNA structures and process them to yield genetic rearrangements (e.g., deletions, inversions, and translocations). Elucidating these pathways will be particularly relevant to cancer biology since, as predicted [10,76], non-B DNA conformations may play an active role in inducing mutations in cancer genomes.

Several families of noncoding RNAs are synthesized in the cell, some of which interact with duplex DNA to form triplex structures that play critical functional roles. Thus, triplex interactions enable lncRNAs to enhance their transcription regulatory activity by providing a scaffolding platform for the efficient delivery of site-specific epigenetic modifications. Given the large fraction of genomic DNA being transcribed, it will be interesting to assess how widespread the use of DNA–RNA tertiary interactions are in gene regulation in vivo.

Most recent work has revealed that triple interactions between and within RNA molecules serve critical functions to cellular processes such as splicing, telomerase activity, and protein recoding, an activity that appears to extend beyond viruses. However, many details remain to be clarified concerning the exact mechanisms through which these tertiary structures elicit their roles, particularly in relation to telomerase function.

Our discussion has focused on the emerging roles of triplex nucleic structures as effectors of biological activity and, as a result, has not entertained other areas of investigation, such as triplex-forming oligonucleotides (TFOs), in which triplex interactions are being pursued as a means to artificially alter gene structure and function, such as gene expression regulation. Thus, in the context of elucidating mechanisms that lead to triplex-induced genetic instabilities, it will be critical to assess whether differences exist in the repair pathways involved in processing endogenous (i.e., H-DNA) and exogenous (i.e., TFO-derived) triplex structures.

In summary, recent years have witnessed an expansion in the research on triplex base-pair interactions, from studies predominantly oriented toward DNA–DNA interactions to investigations aimed at elucidating DNA–RNA and RNA–RNA triplexes. It is now critical to focus on how these structures are being recognized, utilized functionally, and processed in cells.
